# Magnitude of obesity alone does not alter the alveolar lipidome

**DOI:** 10.1152/ajplung.00112.2024

**Published:** 2024-09-10

**Authors:** William G. Tharp, Carolyn R. Morris, Yulica Santos-Ortega, Calvin P. Vary, S. Patrick Bender, Anne E. Dixon

**Affiliations:** ^1^Department of Anesthesiology, University of Vermont, Burlington, Vermont, United States; ^2^Department of Medicine, University of Vermont, Burlington, Vermont, United States; ^3^Center for Molecular Medicine, MaineHealth Institute for Research, Scarborough, Maine, United States; ^4^University of Maine Graduate School of Biomedical Science and Engineering, University of Maine, Orono, Maine, United States; ^5^School of Medicine, Tufts University, Boston, Massachusetts, United States

**Keywords:** bronchoalveolar lavage, lipidome, lung, obesity, surfactant

## Abstract

Obesity may lead to pulmonary dysfunction through complex and incompletely understood cellular and biochemical effects. Altered lung lipid metabolism has been identified as a potential mechanism of lung dysfunction in obesity. Although murine models of obesity demonstrate changes in pulmonary surfactant phospholipid composition and function, data in humans are lacking. We measured untargeted shotgun lipidomes in two bronchoalveolar lavages (BALs) from apical and anteromedial pulmonary subsegments of 14 adult subjects (7 males and 7 females) with body mass indexes (BMIs) ranging from 24.3 to 50.9 kg/m^2^. The lipidome composition was characterized at the class, species, and fatty acyl/alkyl level using total lipid molecular ion signal intensities normalized to BAL protein concentration and epithelial lining fluid volumes. Multivariate analyses were conducted to identify potential changes with increasing BMI. The alveolar lipidomes contained the expected composition of surfactant-associated phospholipids, sphingolipids, and sterols in addition to cardiolipin and intracellular signaling lipid species. No significant differences in lipidomes were detected between the two BAL regions. Though a small number of lipid species were associated with BMI in multivariate analyses, no robust differences in lipidome composition or specific lipid species were identified over the range of body habitus. The magnitude of obesity alone does not substantially alter the alveolar lipidome in patients without lung disease. Differences in lung function in patients with obesity and no lung disease are unlikely related to changes in alveolar lipid composition.

**NEW & NOTEWORTHY** Altered lung lipid metabolism has been identified as a potential mechanism of lung dysfunction in obesity, but data in humans are lacking. We measured the alveolar lipidome in bronchoalveolar lavages from subjects with healthy lungs with a wide range of body mass index. There were no differences in lipidome composition in association with the magnitude of obesity. In patients with healthy lungs, obesity alone does not alter the alveolar lipidome.

## Graphical abstract

**Figure:**
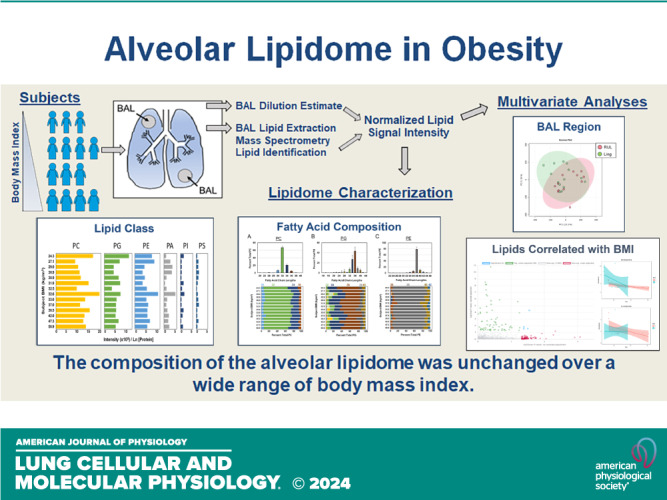

## INTRODUCTION

Obesity leads to pathological alterations in the pulmonary system including restrictive lung physiology, increased airway hyperreactivity, chronic low-grade inflammation, and elevated metabolic demand (1–3). Increasing adiposity contributes to pulmonary dysfunction through a complex and incompletely understood mix of biochemical, cellular, and mechanical effects. Lipid metabolism in the lung is tightly regulated, producing and recycling lipid species necessary to maintain the pulmonary surfactant that lowers the surface tension at the air-liquid interface in the alveoli and serves as a physical and immunological barrier (4–6). The effect of obesity on lung lipid metabolism in humans is not known. Murine models of diet-induced obesity demonstrate changes in surfactant function and phospholipid composition (3, 7, 8). These biochemical changes were associated with altered lung mechanics and increased susceptibility to viral infection, suggesting altered alveolar lipid metabolism may be a pathological mechanism in pulmonary dysfunction of obesity (7, 8).

Alveolar lipids are primarily part of the pulmonary surfactant, which is a combination of glycerophospholipids, sphingolipids, neutral lipids, and four specific proteins produced and secreted by alveolar type 2 cells (4, 5, 9). The complex mixture maintains low alveolar surface tension under dynamic conditions due to the biophysical properties of the phospholipids and their interactions with three of surfactant proteins. Glycerophospholipids typically constitute 85% of the surfactant mass with phosphatidylcholine (PC) species accounting for ∼75% of all phospholipids. The fully saturated dipalmitoyl-PC (DPPC; PC 16:0/16:0) is the predominant species, comprising 40%–50% of the surfactant phospholipids. The straight palmitoyl fatty acid (FA) chains allow formation of a tightly packed biofilm that retains low surface tension through the range of breathing pressures (9, 10). Unsaturated PC species account for another 20%–30% and phosphatidylglycerol (PG) species around 10%–15%. Minor surfactant lipids include phosphatidylinositol (PI), phosphatidylethanolamine (PE), and sphingomyelin (SM), with each class accounting for 2%–4% of all phospholipids. Neutral lipids (e.g., cholesterol) make up 5% of the total surfactant mass. The length and saturation of the fatty acid chains in the phospholipids largely determine the fluidity and surface tension-reducing properties of surfactant. The anionic phospholipids (PG and PI) act as necessary cofactors, integrating the hydrophobic surfactant protein-B (SP-B) and surfactant protein-C (SP-C) into the lipid monolayer. Alterations in phospholipid metabolism and transport can profoundly affect surfactant composition and function (4, 5, 11). Dietary fat intake can influence the fatty acyl composition of surfactant phospholipid species in normal-weight rats (12). Diet-induced obesity in mice reduces the overall diversity of lipid species, shifts phospholipid composition toward longer, unsaturated fatty acids, and alters surfactant function dependent on the diet formulation (3, 7, 8).

Beyond the mechanical properties of surfactant, altered alveolar lipid species have also been implicated in fibrotic diseases, pneumonia, lung injury, and the acute respiratory distress syndrome (4, 5). The loss of myristic (FA 14:0) acid-containing phospholipids in mice on high-fat diets increased susceptibility to SARS-CoV-2 infection (7). Increased cardiolipin species and changes in phospholipid abundance (e.g., PG/PI ratio) have been observed in pneumonia and lung injury (4, 13–15). Similarly, sphingolipids and ceramide species are increased by injury, inflammation, and hypoxia (16). Although the discrete roles of these lipid species in alveolar signaling pathways and as biomarkers of pulmonary disease and injury have yet to be elucidated, altered lipid metabolism is a hallmark of obesity. Increased ceramide signaling and changes in mitochondrial cardiolipin content have been implicated in promoting tissue inflammation and cellular metabolic dysfunction in obesity (17).

We hypothesized that increasing obesity leads to progressive changes in lung lipid metabolism and surfactant composition. Accordingly, we measured the lipidome in bronchoalveolar lavages (BALs) from two pulmonary subsegments in a cohort of subjects with a wide range of body mass indexes to understand the impact of obesity on the alveolar lipidome and pulmonary surfactant composition.

## MATERIALS AND METHODS

### Clinical Study and Ethics Statement

Adult patients undergoing robotic-assisted, laparoscopic surgery at the University of Vermont Medical Center were enrolled in an IRB-approved (M18-0640) cross-sectional study of body habitus and intraoperative lung injury. All participants provided written informed consent. Subjects presented for hysterectomy or prostatectomy for cancer, but were not taking chemotherapeutics. Exclusion criteria included pulmonary diseases; smoking in the last 6 mo; ≥20 pack year smoking history; cardiac, hepatic, or renal dysfunction; and use of chronic anti-inflammatory medications. Demographic and anthropometric data were collected from the medical record and in the preoperative ward.

### Bronchoalveolar Lavage Sampling and Processing

**Figure 1.**
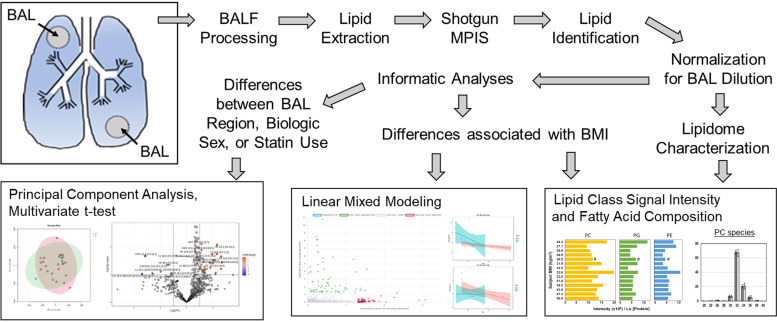
Study schematic. Bronchoalveolar lavages (BAL) were obtained from right upper lobe and superior lingula pulmonary subsegments. The BAL fluid (BALF) was processed to remove cells and debris. Lipids were extracted, applied to untargeted multiple precursor ion scanning (MPIS) mass spectrometry, and lipids were identified from the ion fragments. Ion intensity data were normalized to BAL protein and epithelial lining fluid volumes and used to characterize the lipidome composition. Multivariate informatics methods were used to investigate the differences in the lipidomes between BAL regions, sex, statin use, and for changes associated with body mass index (BMI).

After intubation, fiberoptic bronchoscopy (Ambu) was used to obtain BAL from the right upper lobe (RUL) and superior lingula (Fig. 1). Either 75 or 100 mL of warm saline were instilled and collected under low suction pressure. BAL fluid (BALF) was immediately centrifuged at 500 *g*, decanted, aliquoted, and frozen at −80°C until analysis. Total protein was measured using protein assay (Bio-Rad). Urea was measured by enzymatic assay (Abcam). The epithelial lining fluid (ELF) volume was calculated from the BAL and blood urea concentrations as follows:

$${\text{Volume}}_{\text{ELF}}=\frac{\left[{\text{Urea}}_{\text{BALF}}\right]}{\left[{\text{Urea}}_{\text{Plasma}}\right]}\times \frac{{\text{Recovered Volume}}_{\text{BALF}}}{100}$$

This yields the ELF volume in mL recovered/100 mL BALF (18, 19). Hyaluronic acid and thrombin anti-thrombin complex (TaTc) were quantified by ELISA (R&D Systems; Biomatik) (20).

### Lipid Extraction

Lipids were isolated from 500 µL of BALF using a modified Bligh and Dyer protocol (21, 22). In brief, samples were vacuum concentrated, sonicated, and mixed with methanol (MeOH; Themo Fisher) and dichloromethane (DCM; Sigma Aldrich). Lipids were phase-separated by the addition of water and DCM. The DCM organic phase was removed and dried under nitrogen and partial vacuum. Lipid extracts were dissolved in lipid load solution: MeOH/DCM (50:50, vol/vol) containing LC/MS grade 10 mM ammonium acetate (NH_4_Ac; Fisher Chemical).

### Mass Spectrometry

Unbiased multiple precursor ion scanning (MPIS) lipidomic analyses were performed by direct infusion of sample in lipid load solution (22). A 100 µL sample of a fourfold diluted lipid extract in lipid load solution was delivered by PAL3 System (LEAP Technologies) to a Sciex QTRAP 4,000 mass spectrometer alongside a second isocratic pump (MX Class HPLC pumps, Teledyne SSI), which delivered lipid load solution at 0.25 mL/min during all the runs. Source parameters included gases GS1 at 15 and GS2 at 20, curtain gas at 10, IonSpray voltage 5,300, temperature at 150°C, and collision energy was 50 eV. Each sample was injected twice and positive and negative polarity mode data were acquired simultaneously in serial experiments from a single-sample infusion. Lipids were analyzed in MPIS and the analytical quadrupole Q1. The instrument was controlled and data were processed using Analyst 1.7.3; acquired data were processed with LipidView 1.3beta and MarkerView 1.4, and lipids were identified through peak matching with the LipidView database (Sciex SO) (23, 24). Most likely ratio (MLR)-normalized signal intensity data were used for all subsequent analyses.

### Data Processing and Lipidome Analyses

Data from each run were combined and replicates averaged. Signal intensity data were normalized to Ln[Protein] and to ELF volume and analyzed separately. Relative abundance was determined as a percentage of signal intensity for all lipids species within a lipid class. Distributions of lipid species relative abundance were compared between BAL locations and by BMI. Informatic analyses were conducted on normalized signal intensity data with both the full dataset and with data filtered for presence in >40% of the samples using Metaboanalyst (www.metabolanalyst.ca) (25). These data were further filtered for variance (40% for the full dataset and 10% for the restricted dataset), square root transformed, and Pareto scaled (see Supplemental Data) (22, 26, 27). Differences between BAL regions, biological sexes, and statin use were assessed by principal component analysis (PCA) and multivariate *t* test. BMI-associated differences in lipidomes were assessed by linear mixed modeling. Raw and false-discovery rate (FDR)-corrected *P* values are presented, with corrected *P* < 0.05 indicating significance. Data are presented as means ± standard deviation when the data are normally distributed and as median [interquartile range (IQR)] when nonnormally distributed.

## RESULTS

### Cohort Description and BAL Sampling

We recruited and studied 14 subjects (7 males and 7 females) with an average age of 59.1 ± 7.5 yr and mean BMI of 34.9 ± 7.5 kg/m^2^ with a range of 24.3 to 50.9 kg/m^2^ (Table 1). Five subjects were taking a statin for hyperlipidemia, seven were on hypertension medications, and three were on therapy for type 2 diabetes mellitus.

**Table 1.** Cohort characteristics

	Value
Cohort size (*n*)	14
Sex (m/f)	7/7
Age, yr	59.1 ± 7.5
Height, cm	173.0 ± 7.5
Weight, kg	104.0 ± 21.4
Body mass index (BMI), kg/m^2^	34.9 ± 7.5
BMI range	24.3 to 50.9
Current medications for:	
Hyperlipidemia, yes/no	5/9
Hypertension, yes/no	7/7
Type 2 diabetes mellitus, yes/no	3/11

BAL recovery from the RUL averaged 30 ± 12% (28 ± 12 mL) and recovery from the lingula averaged 36 ± 15% (31 ± 13 mL) (Supplemental Fig. S1*A*). Adequate BAL fluid for lipidomic analysis was obtained from 13 RUL and 14 lingula lavages. Total protein concentrations from the RUL had a median of 83.0 µg/mL [52.2 to 122.0], whereas those from the lingula were 157.2 µg/mL [108.6 to 175.2] (Supplemental Fig. S1*B*). Urea concentrations had a median values of 1.9 µg/mL [1.4 to 2.7] in the RUL and 2.2 µg/mL [1.0 to 5.6] in the lingula, whereas circulating urea concentrations were 270.3 µg/mL [249.9 to 324.8] (Supplemental Fig. S1*C*). One subject did not have a blood sample, so we used the median as their sample. ELF recovery from RUL had a median of 0.68 mL/100 mL BAL [0.44 to 1.08] and was 0.65 mL/100 mL BAL [0.36 to 1.39] from the lingula (Supplemental Fig. S1, *D* and *E*).

Hyaluronic acid concentrations from the RUL averaged 37.7 ± 49.3 ng/mL and those from the lingula averaged 21.1 ± 12.6 ng/mL. No TaTc was detected in any BAL samples. These data indicated no evidence of alveolar disruption or damage to the lung tissues.

### Lipid Detection and Regional Composition by Class

A total of 2,066 lipid subspecies were detected from 15 classes/subclasses (Table 2), including the major surfactant-associated glycerophospholipids [phosphatidic acid (PA), phosphatidylcholine (PC), phosphatidylethanolamine (PE), phosphatidylglycerol (PG), phosphatidylinositol (PI), phosphatidylserine (PS)]; cardiolipins (CL); sphingolipids [sphingomyelin (SM), ganglioside sphingolipids (GD1, GD2, GD3, GM1, GM2, GM3, GT2, and GT3), ceramides (Cer), phosphor-ceramides (CerP), hexoceramides (HexCer, Hex2Cer, Hex3Cer), PI-ceramide (IPC)]; sterols [cholesterol esters (CEs), desmosterol (Des), ergosterol (Erg)]; secondary messenger phospho-PI species (PIP, PIP2, PIP3); phospholipid precursors [cytidine diphosphate-diacylglycerol (CDP-DAG)]; and acylglycerols [diacylglycerol (DAG), monoalkyl-DAG (MADAG), triacylglycerol (TAG)]. Many lipid species had sparse detection over all the samples; 295 species were detected in >40% of samples (Table 2 and Supplemental Fig. S2).

**Table 2.** Lipid classes and species detected

Lipid Class	No. of Species Identified	No. of Species Present in >40% of Samples
PC	106	40
PG	182	44
PE	111	26
PA	191	16
PI	126	14
PS	116	17
PIPs	329	32
CDP-DAG	53	5
CL	429	45
SM	36	7
Sterols	31	5
Ceramides	118	14
Gangliosides	182	19
DAG	28	3
TAG	28	8
Total	2,066	295

CDP-DAG, cytidine diphosphate diacylglycerol; PA, phosphatidic acid; PC, phosphatidylcholine; PE, phosphatidylethanolamine; PG, phosphatidylglycerol; PIP, phosphatidylinositol-phosphates; TAGs, triacylglycerols.

**Figure 2.**
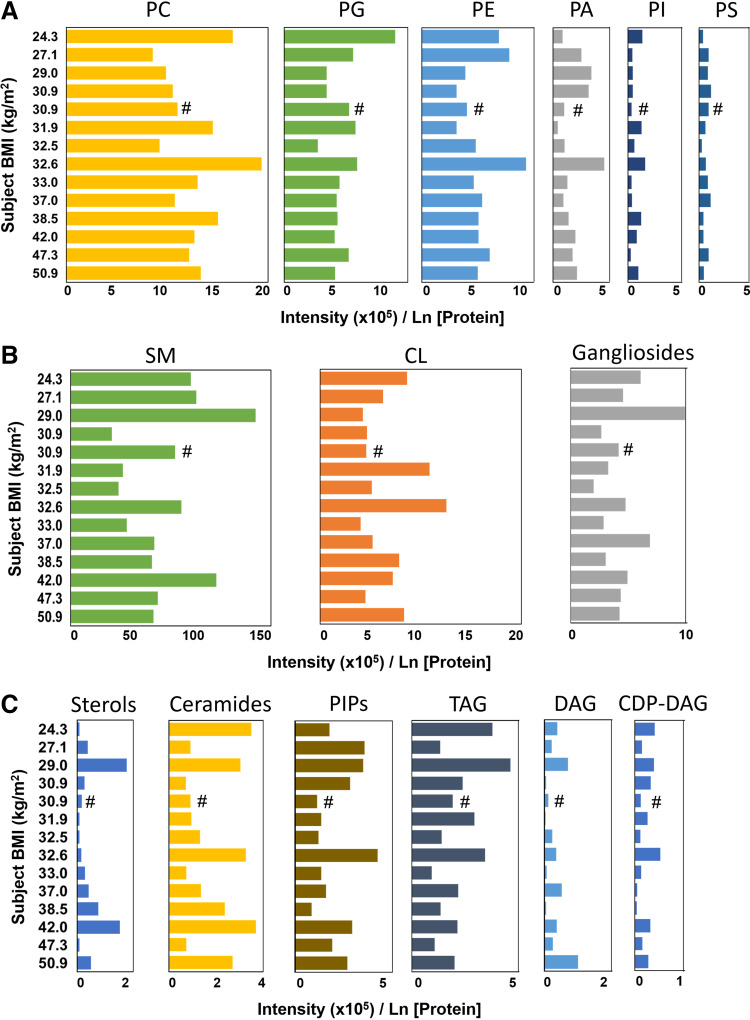
Alveolar lipid class intensity normalized by protein. *A*: the protein-normalized signal intensity for surfactant-associated glycerophospholipids phosphatidylcholine (PC), phosphatidylglycerol (PG), phosphatidylethanolamine (PE), phosphatidic acid (PA), phosphatidylinositol (PI), and phosphatidylserine (PS) arranged by subject body mass index (BMI). *B*: protein-normalized signal intensity for sphingomyelin (SM), cardiolipins (CL), and ganglioside lipids arranged by subject BMI. *C*: protein-normalized signal intensity for sterols, ceramides, PI-phosphates (PIPs), triacylglycerols (TAGs), diacylglycerols (DAG), and cytidine diphosphate DAG (CDP-DAG). Each bar represents the average of the 2 BAL, except for (#), which had only one sample. Note the differences in scale for each panel. Presented as intensity count × 10^5^/natural logarithm [protein].

The total lipid signal intensity in each lipid class did not differ between lavage regions when normalized to protein concentration (Supplemental Fig. S3) or to ELF volume (Supplemental Fig. S4). Lipid signal intensity was not associated with BMI in any lipid class when normalized by protein (Fig. 2) or by ELF volume (Supplemental Fig. S5).

### Lipid Abundance and Composition by Species and Fatty Acid Chain Length

**Figure 3.**
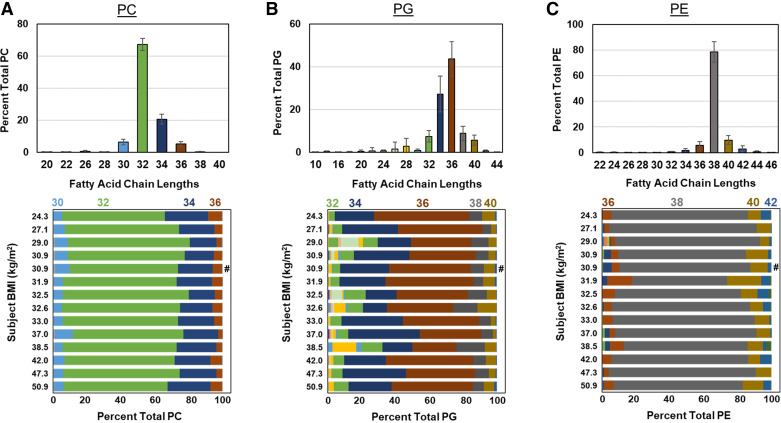
Alveolar phospholipid fatty acid composition. *A*: overall distribution (*top*) of phosphatidylcholine (PC) fatty acid composition and individual distributions (*bottom*) arranged by subject body mass index (BMI). *B*: global distribution (*top*) of phosphatidylglycerol (PG) fatty acid composition and individual distributions (*bottom*). *C*: overall distribution (*top*) of phosphatidylethanolamine (PE) fatty acid composition and individual distributions (*bottom*). Colors in all panels correspond to total fatty acid chain lengths. Individual distributions are the average of the 2 bronchoalveolar lavages (BALs), except for (#), which had only one sample.

**Figure 4.**
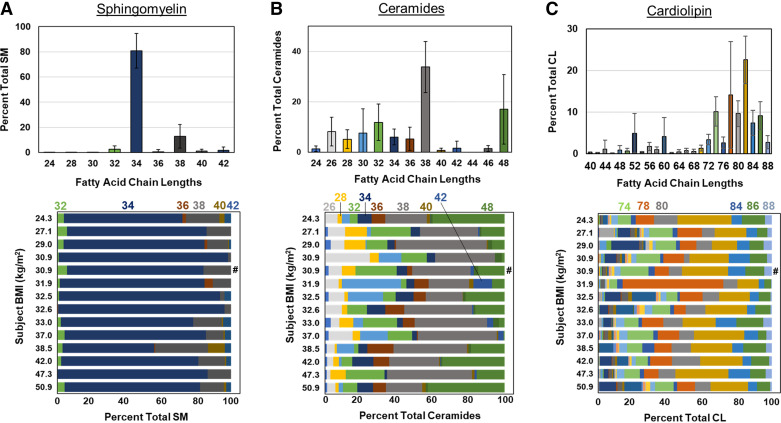
Alveolar lipid fatty acid composition. *A*: overall distribution (*top*) of sphingomyelin (SM) fatty acid composition and individual distributions (*bottom*) arranged by subject body mass index (BMI). *B*: global distribution (*top*) of ceramide fatty acid composition and individual distributions (*bottom*). *C*: overall distribution (*top*) of cardiolipin (CL) fatty acid composition and individual distributions (*bottom*). Colors in all panels correspond to total fatty acid chain lengths. Individual distributions are the average of the 2 bronchoalveolar lavages (BALs), except for (#), which had only one sample.

Examination of the fatty acid chain length distributions of the major phospholipid classes demonstrated the expected predominance of PC 32, PE 38, PG 34, and PG 36 species and no differences were observed with increasing BMI (Fig. 3) or between lavage regions (Supplemental Figs. S6 and S7). The fatty acid composition of SM showed predominance of SM 34 species (Fig. 4*A*). No differences in fatty acid composition were observed for SM, ceramide, or CL species with increasing BMI (Fig. 4) or lavage region (Supplemental Fig. S8), nor were there BMI-related differences in sterol ester, DAG, TAG, or ganglioside composition (Supplemental Figs. S9 and S10). No BMI-related differences in the percentage of saturated or unsaturated fatty acid species were observed (Supplemental Fig. S11).

### Multivariate Analyses of Differences in Alveolar Lipidome by Lavage Region, Sex, and Statin Use

PCA demonstrated near-complete overlap of the 95% confidence areas for the two BAL regions for both normalization methods (Supplemental Fig. S12, *A* and *B*). Similarly, PCA confidence areas overlapped when comparing biologic sex (Supplemental Fig. S12, *C* and *D*) or use of statin medication (Supplemental Fig. 12, *E* and *F*) by both protein and ELF normalization methods. No differences in lipid species between lavage regions or statin use were identified by multivariate *t* test. A single lipid species (PIP 34:4) was absent in all males but present in samples from six of the seven females. No other differences between biological sexes were identified by multivariate *t-*test. Restricting the analyses to lipid species present in ≥40% of the samples obtained the same results (Supplemental Fig. S13). One outlier sample was identified as having excess leverage on the analyses, especially within the ELF normalized data. No changes in the results were noted with the outlier removed (Supplemental Fig. S14).

### Lipid Species Association with Body Mass Index by Multivariate Analyses

Next, we used linear mixed modeling to examine association of lipid species with increasing BMI. For the full dataset normalized to protein, 14 lipid species had linear associations with BMI after controlling for subject, lavage region, and sex, and a subset of 9 lipids were associated with BMI when only controlled for subject (Table 3). Two lipid species (PG 40:6 and GM1 44:0;3) were associated with increasing BMI in ELF-normalized data controlled for subject, lavage region, and sex (Table 4). When the only covariate was subject, another lipid (PG 40:1) was also associated with increasing BMI. Linear modeling of lipids present in ≥40% of samples found no associations of any lipid species with BMI under either normalization method. Analyses with the outlier removed narrowed the lipids associated with BMI to seven species for data normalized by protein (Table 3), but the ELF-normalized data were unchanged (Table 4).

**Table 3.** Correlation of lipid species with body mass index in protein-normalized data

Lipid Species	Correlation Coefficient	Raw *P* Value	FDR-Adjusted *P* Value
*Full dataset, covariates: subject, lavage region, sex*			
PG 36:6	0.685	2.8E-09	4.2E-06
GD2 24:0;3	0.522	5.4E-08	4.0E-05
PIP 28:7	0.613	1.6E-06	8.0E-04
CL 70:4	0.294	2.6E-06	8.2E-04
GM1 44:0;3	0.556	2.8E-06	8.2E-04
PS 40:3	0.324	1.5E-05	0.004
PG 40:6	0.410	2.7E-05	0.006
PE 34:3	0.301	4.0E-05	0.007
PE 36:0	0.293	4.3E-05	0.007
CL 84:7	0.309	7.8E-05	0.012
GM1 46:4;2	0.530	8.7E-05	0.012
PG 40:1	0.269	1.8E-04	0.023
CL 86:10	0.346	2.3E-04	0.025
CL 78:14	0.311	2.3E-04	0.025
*Full dataset, covariates: subject*			
PG 36:6	0.70082	8.8E-10	7.9E-07
GD2 24:0;3	0.53635	2.2E-08	9.8E-06
GM1 44:0;3	0.52913	2.2E-06	6.4E-04
CL 70:4	0.30086	2.9E-06	6.4E-04
CL 84:7	0.32644	3.3E-05	0.006
PG 40:6	0.37869	4.7E-05	0.007
CL 78:14	0.30847	1.4E-04	0.018
GM1 46:4;2	0.47718	1.7E-04	0.018
PG 40:1	0.25296	2.9E-04	0.029
*Restricted dataset, covariates: subject, lavage region, sex*			
None			
*Restricted dataset, covariates: subject*			
None			

**Table 4.** Correlation of lipid species with body mass index in epithelial lining fluid-normalized data

Lipid Species	Correlation Coefficient	Raw *P* Value	FDR-Adjusted *P* Value
*Full dataset, covariates: subject, lavage region, sex*			
GM1 44:0;3	0.934	6.1E-07	5.4E-04
PG 40:6	0.656	2.0E-05	0.009
*Full dataset, covariates: subject*			
PG 40:6	0.626	1.8E-05	0.016
GM1 46:4;2	0.785	3.9E-05	0.018
PG 40:1	0.442	1.5E-04	0.044
*Restricted dataset, covariates: subject, lavage region, sex*			
None			
*Restricted dataset, covariates: subject*			
None			

## DISCUSSION

This study found that the alveolar lipidome, sampled by bronchoalveolar lavage, is essentially unchanged by increasing magnitude of obesity. We observed no broad changes in the relative abundance of alveolar lipid classes or fatty acid composition of alveolar lipid species over the range of body habitus. We found no differences in lipidomes from apical or anteromedial lavages. Two lipid species, PG 40:6 and GM1 44:0;3, were consistently associated with increasing BMI in lipid data normalized to either protein concentrations or ELF volumes in the full dataset, but no individual lipid species were associated with BMI in more stringent analyses. Our data are consistent with previous studies of surfactant phospholipid composition from healthy volunteers in terms of relative lipid class abundances and homogeneity of surfactant from different lung regions (10, 28). It is important to note that we did not isolate surfactant aggregates so the lipidome also contains lipids that are not in the surfactant. We detected a substantial abundance of CL species in addition to lipids canonically associated with intracellular signaling (e.g., PIP, DAG, and TAG), in the absence of alveolar injury. Overall, our data indicate that the magnitude of obesity does not substantially alter the alveolar lipidome or surfactant composition in patients without lung disease.

Mice made obese using high-fat diets demonstrated reduced myristic acid (FA 14:0)-containing PC and PG lipid species in BALF, measured by absolute quantification (7, 8). Mice on both high-fat and high-carbohydrate diets demonstrated increased oleic acid (FA 18:1) and linoleic acid (FA 18:2)-containing PG species in BALF and impaired surfactant spreading (8). Lipid profiles from induced sputum of healthy volunteers suggested an effect of BMI on the enrichment of non-DPPC phospholipids measured against PC internal standards (6). We identified differences in PG 40:6 and GM1 44:0;3 associated with BMI in samples from both normalization methods in the full lipid dataset. Although several more lipid species were associated with BMI in the full dataset normalized to protein, no individual lipid species were associated with body habitus in the analyses of the 40% restricted datasets normalized by either method.

Targeted, absolute quantification of surfactant phospholipid dynamics shows that the fatty acid composition of PC rapidly responds to dietary intake (12, 28). Normal-weight rats fed low-fat diets (<15% of energy from fat) enriched with specific FA species for only 7 days showed shifts in the FA composition of PC species (12). When the diets were enriched for myristic acid (FA 14:0), PC 28:0 and PC 30:0 species increased and PC 32:0 and PC 32:1 decreased; when the diets were enriched with linoleic acid (FA 18:2), PC 32:0 and PC 34:2 species increased and PC 32:1 species decreased. Human surfactant turnover is similarly rapid, with 50% incorporation of deuterated methyl-D_9_-choline into alveolar PC species at 48 h (28). Taken together, the rapid turnover and dynamic responsiveness to dietary intake suggest that the magnitude of obesity alone does not result in substantial changes in lung lipid metabolism, at least in the bronchoalveolar compartment. Rodents fed highly regimented diets demonstrate changes in surfactant lipid composition, but the specific changes appear related to the diet composition (e.g., high carbohydrate vs. high fat). Thus, our subjects, who were not on strictly regimented diets, do not robustly demonstrate surfactant lipid composition changes.

The abundance of CL and canonical intracellular signaling lipids led us to test for evidence of existing alveolar injury or damage during the BAL. We found no evidence of injury or alveolar-capillary leak in our samples: total protein, urea, and hyaluronic acid concentrations were within normal ranges and TaTc was undetectable. Although CL is typically mitochondrial, its presence has been documented in BALF under normal and pathological conditions (4, 13, 15). Furthermore, CL is also known as di-phosphatidylglycerol (PG) and is produced in a series of reactions involving CDP-DAG and PG (14, 15). Numerous individual CL and PG species were associated with BMI in analyses of the full dataset normalized by protein (Table 3). Given the mixed changes in PG species found in prior studies and our data, we posit that the pathways involved in CDP-DAG/PG/CL synthesis and degradation may respond dynamically to metabolic changes. The presence of canonically intracellular or signaling lipids, such as PIP and DAG species, was also intriguing, after ruling out gross cellular injury. Phosphorylated PI species are generally associated with g-protein-coupled receptor signaling and the intracellular PI3K-Akt pathway, but can be released as an extracellular signal in apolipoproteins and extracellular vesicles (29). The presence of CL, PIP, and DAG species may come from these lipid-laden particles. One other source of CL or intracellular lipids may be bacteria or fungi. We detected ergosterol species in six subjects, which could represent the endogenous provitamin for vitamin D_2_ (ergocalciferol) or fungal cell membrane components, the former being more likely.

Ceramides and sphingolipid metabolism have been implicated in lipotoxic adipose tissue dysfunction (17). Ceramides are both sphingolipid precursors and degradation products. Accumulation of ceramide species inhibits insulin signaling along the Akt-PKB pathway and these lipids are strong biomarkers of metabolic disease. Altered pulmonary sphingolipid metabolism has been documented in acute respiratory distress syndrome, infections, vascular remodeling, and chronic obstructive pulmonary disease (16, 30). Although several sphingolipids were associated with BMI in multivariate analyses of the full dataset, there does not appear to be a robust effect of obesity alone on lung sphingolipid levels.

This study has some limitations. First, our study population is small and contains only one subject with BMI < 25 kg/m^2^ and two subjects with BMI 25–29.9 kg/m^2^. However, the cohort has wide range of BMI and there is a lack of clear trajectories with increasing BMI. Given these two facts, it is reasonable to conclude that the magnitude of obesity alone has no substantial effect on the alveolar lipidome, but it is possible that differences identified in the least stringent analyses may be significant with a larger cohort. These data clearly need to be replicated. Second, BMI was unevenly distributed by sex, female subjects had higher BMI than our male subjects. However, we found no clear effect of biologic sex on alveolar lipidomes beyond a single PIP 34:4 species. Taken in context with a lack of effect from BMI, the uneven distribution is not confounding the interpretation. Third, we did not perform absolute quantification of lipids in these lipidomes. Each lipid class has different extraction and ionization kinetics and thus the molecular signal intensities are not directly comparable between classes. This may also affect the complex lipids, such as the polar GM lipids, limiting accurate comparisons between species. However, because the gangliosides are associated with BMI in the least stringent analyses, we are reporting these data as they may form the basis of future targeted analyses. Fourth, we did not isolate surfactant aggregates, but assessed the entire alveolar lipidome. We cannot definitively say which lipid species are part of the surfactant and which are involved in extracellular signaling or other molecular pathways.

### Conclusions

In this cross-sectional study, we found no substantial effect of increasing obesity on the composition of the alveolar lipidome derived from two regionally distinct BAL. Magnitude of obesity alone does not alter alveolar lipid metabolism or composition. Differences in lung function in patients with obesity and no lung disease are unlikely related to alveolar lipid composition.

## DATA AVAILABILITY

Data will be made available upon reasonable request.

## SUPPLEMENTAL MATERIAL

10.6084/m9.figshare.26771044 Supplemental Figs. S1–S14: https://doi.org/10.6084/m9.figshare.26771044.

## GRANTS

W.G.T. was supported by a mentored research training grant from the International Anesthesia Research Society and a Loan Repayment Award from the National Heart, Lung, and Blood Institute (L30HL149005-01). The MaineHealth Institute for Research Proteomics and Lipidomics Core is supported by Center of Biomedical Research Excellence (COBRE) in Mesenchymal and Neural Regulation of Metabolic Networks (P20GM121301) and the Northern New England Clinical and Translational Research Network Programs (U54GM115516) from the National Institute of General Medical Sciences.

## DISCLOSURES

No conflicts of interest, financial or otherwise, are declared by the authors.

## AUTHOR CONTRIBUTIONS

W.G.T., S.P.B., and A.E.D. conceived and designed research; W.G.T., Y.S.-O., C.P.V., and S.P.B. performed experiments; W.G.T., C.R.M., Y.S.-O., and C.P.V. analyzed data; W.G.T., C.R.M., C.P.V., and A.E.D. interpreted results of experiments; W.G.T. prepared figures; W.G.T. drafted manuscript; W.G.T. edited and revised manuscript; W.G.T., C.R.M., Y.S.-O., C.P.V., S.P.B., and A.E.D. approved final version of manuscript.
